# A Clue for the Hen and Egg Question: The Simultaneous Formation of Uracil and Amino Acids Under Simulated Hadean Conditions

**DOI:** 10.3390/life16040624

**Published:** 2026-04-08

**Authors:** Christian Seitz, Denis Schuldeis, Konstantin Vogel, Wolfgang Eisenreich, Claudia Huber

**Affiliations:** Strukturelle Membranbiochemie, Bayerisches NMR Zentrum, Technische Universität München, Lichtenbegrstr. 4, 85748 Garching, Germany; c.seitz@tum.de (C.S.); denis.schuldeis@tum.de (D.S.); ge42gik@tum.de (K.V.); wolfgang.eisenreich@mytum.de (W.E.)

**Keywords:** uracil, amino acids, acetylene, carbon monoxide, cyanide transition metal sulfides, hydrothermal conditions, origin-of-life

## Abstract

The origin of life is commonly discussed within two competing conceptual frameworks: the metabolism-first and information-first hypotheses. While each emphasizes a different defining property of early life, modern biochemistry reveals a fundamental interdependence between metabolic processes and genetic information transfer, leading to a persistent chicken-and-egg problem. In this study, we investigate a prebiotically plausible reaction system that enables the concurrent formation of molecular precursors associated with both frameworks. Under simulated Hadean hydrothermal conditions, acetylene, ammonia, cyanide, and carbon monoxide were reacted in aqueous solution in the presence of transition metal sulfides. Using gas chromatography–mass spectrometry combined with stable isotope labeling, we demonstrate the simultaneous formation of the nucleobase uracil and the amino acids alanine and aspartic acid. Isotopic incorporation patterns allow reconstruction of the underlying reaction pathways and confirm the contribution of all starting materials to product formation. While amino acids are produced continuously over the observed period in significantly higher yields than uracil, uracil formation exhibits a pronounced time-dependent maximum after three days. Variations in pH, reaction time, and metal sulfide catalysts modulate product yields but do not prevent the parallel emergence of both molecular classes. These findings support a scenario in which proto-metabolic chemistry and molecular precursors of genetic information could have arisen simultaneously within a shared geochemical setting. The results provide experimental support for a coupled origin of metabolism and transcriptional building blocks, offering a potential resolution to the dichotomy between metabolism-first and information-first models of early life.

## 1. Introduction

The origin of life remains one of the most profound and challenging questions in modern science, situated at the intersection of chemistry, biology, and physics. Understanding how non-living matter transitioned into self-sustaining, evolving systems is essential for reconstructing the early history of life on Earth and for assessing the potential for life elsewhere in the universe. Despite substantial advances in experimental and theoretical research, there is still no consensus on the sequence of events that led to the emergence of the first living systems.

Two major conceptual frameworks dominate contemporary discussions on the origin of life [1,2]: the metabolism-first [3,4,5,6] and the information-first hypotheses [7,8,9]. These frameworks differ primarily in their assumptions about which defining feature of life—self-sustaining chemical organization or genetic information processing—emerged first.

The information-first hypothesis emphasizes the early emergence of genetic information transfer before the evolution of full biological replication and translation systems. In this view, the ability to transcribe genetic information—rather than to replicate it with high fidelity or translate it into proteins—played a central role in the earliest stages of life. In modern biology, transcription refers to the process by which genetic information stored in DNA is copied into RNA. In an origin-of-life context, however, information-first models typically assume a pre-DNA world, often closely related to the RNA world hypothesis. In such scenarios, RNA molecules acted both as information carriers and as functional molecules, comparable to ribozymes [10,11]. Early replication-like processes may have involved the copying of RNA templates into complementary RNA strands, even if these processes were inefficient and error-prone. The key idea behind *information-first* is that informational continuity could arise before accurate self-replication. Early protocells may have contained RNA molecules that were repeatedly transcribed from templates, allowing certain sequences to persist longer than others. This persistence would enable a primitive form of selection, favoring RNA sequences that were more stable, more easily transcribed, or more functionally active. One advantage of the information-first perspective is that it reduces the initial complexity required for life to begin. High-fidelity replication is chemically demanding, whereas simple template-directed RNA synthesis could plausibly occur under prebiotic conditions. Even low-accuracy replication could generate diversity, which is essential for evolutionary processes to begin.

In contrast, the metabolism-first hypothesis proposes that life originated not from genetic information, but from self-sustaining chemical reaction networks. According to this view, organized metabolism preceded the emergence of genetic systems such as RNA or DNA. Life is thus conceptualized as a dynamic chemical process rather than as an information-based system. Early Earth environments provided energy sources and chemical gradients—such as those found near hydrothermal vents—that could drive spontaneous reactions among simple molecules. These reactions may have formed autocatalytic networks, reaction products facilitated further reactions, allowing the system to persist and evolve without genetic instructions. A major strength of the metabolism-first hypothesis is that it addresses how complex biochemical pathways could arise in the absence of pre-existing genetic information. Basic metabolic cycles, similar to parts of the modern citric acid cycle, may have emerged through geochemical processes catalyzed by mineral surfaces [12,13]. These cycles could concentrate matter, store energy, and generate increasingly complex organic molecules [14].

From the perspective of modern biochemistry, however, the transfer of information in the form of transcription and metabolism are deeply interconnected [15]. Metabolism provides the chemical and energetic foundation required for transcription. The synthesis of RNA depends on metabolic pathways that generate nucleotides, ribose sugars, phosphate groups, and energy carriers such as ATP and GTP. Central metabolic pathways, including glycolysis, citrate cycle, pentose phosphate pathway, and amino acid biosynthesis, supply both the building blocks and reducing power necessary for RNA production [16]. Without a functioning metabolism, transcription cannot proceed. At the same time, transcription exerts control over metabolism by regulating the expression of metabolic enzymes. Cells adjust transcriptional programs in response to nutrient availability, energy status, and environmental stress [17]. For example, changes in metabolite concentrations can activate transcription factors or RNA-based regulatory elements, leading to increased or decreased transcription of genes encoding metabolic enzymes [18]. This allows metabolism to adapt dynamically to changing conditions [17,19]. Metabolites themselves often act as signaling molecules that directly influence transcription. Small molecules such as sugars, amino acids, lipids, and metal ions can bind transcription factors or riboswitches, altering gene expression [20,21,22]. In this way, metabolic state is translated into transcriptional responses, creating feedback loops that maintain cellular homeostasis [19]. This interdependence gives rise to a persistent chicken-and-egg problem: neither process can function independently in contemporary biological systems. One possible resolution is that both characteristics emerged simultaneously within a shared geochemical context. Here, we demonstrate that under conditions inspired by Wächtershäuser’s iron–sulfur world hypothesis, both the nucleobase uracil—which is a building block of the nucleotide uridine, and can therefore be seen as an early representative of the information first hypothesis—and amino acids such as alanine and aspartic acid—key components of metabolism-first scenarios—can form concurrently. Using stable isotope labeling, we show that these molecular classes arise side by side from a common set of simple precursors under simulated Hadean hydrothermal conditions in which early life may have emerged in the vicinity of volcanic exhalations [14] or hydrothermal vent systems [23].

In the investigated reaction network, acetylene and carbon monoxide served as the primary carbon sources. Acetylene is considered to have been readily available on the early Earth, as it can be generated through volcanic processes [24] and by the hydrolysis of calcium carbide [25]. In addition, acetylene plays a key role in interstellar chemistry [26,27], where it acts as a precursor for small hydrocarbons, polyenes, and aromatic compounds. These properties make acetylene a versatile and chemically plausible carbon feedstock for early biochemical evolution [28]. Consistent with this, isotopic labeling patterns in our experiments clearly identify acetylene as the dominant contributor to the carbon backbone of the observed products. Carbon monoxide, the second carbon source in the network, is equally plausible in a prebiotic environment. Firstly, CO is formed during the degassing of the Earth’s interior [29]; secondly, it is produced during the reaction of methane with water. The equilibrium of this reaction shifts in favor of CO at higher temperatures, conditions that are thought to have prevailed in the early stages of Earth’s history [30].

Nitrogen was supplied in the form of cyanide and ammonia, both of which are well justified in the proposed prebiotic context. Cyanide is widely regarded as a central prebiotic molecule, capable of giving rise to a broad range of precursors relevant to both proteins and nucleic acids [31,32,33]. Its availability on the early Earth is supported by multiple formation pathways, including photochemical reactions [34], high-temperature processes during asteroid impacts [35], electrical discharges [36], and cometary delivery [37]. Elevated cyanide concentrations in aqueous environments further lead to the formation of formamide, which decomposes at higher temperatures into ammonia and formic acid [38]. Additionally, ammonia may have been generated via the reduction of nitrate driven by FeS/H_2_S systems [39]. In this scenario, nitrate could have formed from atmospheric N_2_ and CO_2_ by electric discharges under oxygen free conditions [40] and subsequently dissolved in the ancient ocean.

We further show that the transition metals iron, nickel, and cobalt, as well as their mixtures, can all function as effective catalysts in these prebiotic reactions. While the individual metals promote product formation to different extents, their general catalytic competence suggests that a diversity of biochemical building blocks could have emerged from geochemically heterogeneous environments. This notion is consistent with the natural abundance of these metals on the early Earth, where iron, nickel, and cobalt are commonly found in the crust [41,42]. Their relevance is further underscored by extant biochemistry: iron plays a central role in redox-active Fe–S clusters and oxygen transport in hemoglobin, nickel is essential for hydrogen activation and functions as a key component of cofactor F_430_ in methanogenesis, and cobalt is incorporated into coenzyme B_12_, which is required for enzymatic reactions in amino acid metabolism.

## 2. Materials and Methods

All chemicals were purchased from Sigma Aldrich GmbH (Steinheim, Germany) in the highest purity available. Acetylene 2.6 (acetone free) was purchased from Linde AG (Pullach, Germany), and CO 2.5 and argon 4.6 were purchased from Westfalen AG (Münster, Germany).

Experiments were performed as published previously [43,44]. More specifically, in a typical run (run 1, Table S1), a 125 mL glass serum bottle was charged with 1.0 mmol NiSO_4_·6 H_2_O, 1.0 mmol NH_4_Cl, 1.0 mmol KCN and closed with a gas-tight silicon stopper.

The bottle was evacuated three times and filled with argon, finally resulting in a de-aerated state. Subsequently, the bottle was filled with argon-saturated water, 1 M Na_2_S solution and 1 M NaOH solution, resulting in a total reaction volume of 5 mL. In this mixture, a precipitate of black NiS is immediately formed due to its low solubility constant of 1 × 10^−22^ [45,46] in aqueous solution. Finally, 60 mL of acetylene gas and 60 mL of CO were added. Liquids and gases were injected by using gas-tight syringes. The freshly precipitated NiS (NiSO_4_ + Na_2_S → NiS↓ + Na_2_SO_4_) acted as a putative transition metal catalyst for the reaction and the molar variations of Na_2_S to NiSO_4_ resulted in free sulfide ions in the solution. Reactions were carried out at 105 °C. pH variations were achieved through the addition of different volumes of NaOH solution (1 M), H_2_SO_4_ solution (1 M) or solid Ca(OH)_2_. For safety reasons (danger of explosion) and for technical reasons, the reactions were carried out at low gas pressure. Bottles were filled with ~1 bar at room temperature, leading to a total absolute pressure of ~2.5 bar at 105 °C. After the defined reaction time the reaction mixture could cool down and 1 mL was transferred into a test tube, freeze-dried and derivatized with 0.5 mL acetonitrile and 0.5 mL MTBSTFA at 70 °C for one hour.

Stable isotope precursors (^13^CO, ^13^C_2_-acetylene, KC^15^N and ^15^NH_4_Cl) were used to elucidate the composition of the products. ^13^CO, KC^15^N and ^15^NH_4_Cl were directly added instead of their analogs. ^13^C_2_-Acetylene gas was obtained by adding tetra-*n*-butylammonium fluoride (TBAF) to solid ^13^C_2_-(trimethylsilyl)acetylene in an evacuated serum bottle via a syringe. The resulting ^13^C_2_-acetylene gas was then used for experiments.

GC-MS analysis was performed with a GC-2010, coupled with MS-QP2010 Ultra (Shimadzu GmbH, Duisburg, Germany) with a 30 m × 0.25 mm × 0.25 µm fused silica capillary column (Equity TM5, Supelco, Bellefonte, PA, USA) and an AOC-20i auto injector, Shimadzu GmbH, Duisburg, Germany.

Temperature program and settings for silylated products: 0–6 min at 90 °C; 6–25 min at 90–310 °C, 10 °C/min; injector temperature: 260 °C; interface temperature: 260 °C; column flow rate: 1 mL/min; scan interval: 0.5 s; and injection volume 0.5 μL.

Peak assignment was achieved by comparison with the retention times and mass spectra of purchased reference compounds, as well as with data from the National Institute of Standards and Technology (NIST) spectral library.

Quantification was performed by external calibration using a solution of uracil with different concentrations. Runs without a transition metal compound or with argon instead of acetylene were performed for comparison.

## 3. Results

To elucidate the contribution of individual starting materials to product formation, isotopic labeling experiments were conducted using ^13^C- and ^15^N-labeled precursors. Incorporation of labeled atoms resulted in predictable mass shifts, allowing direct identification of precursor–product relationships.

**Figure 1:**
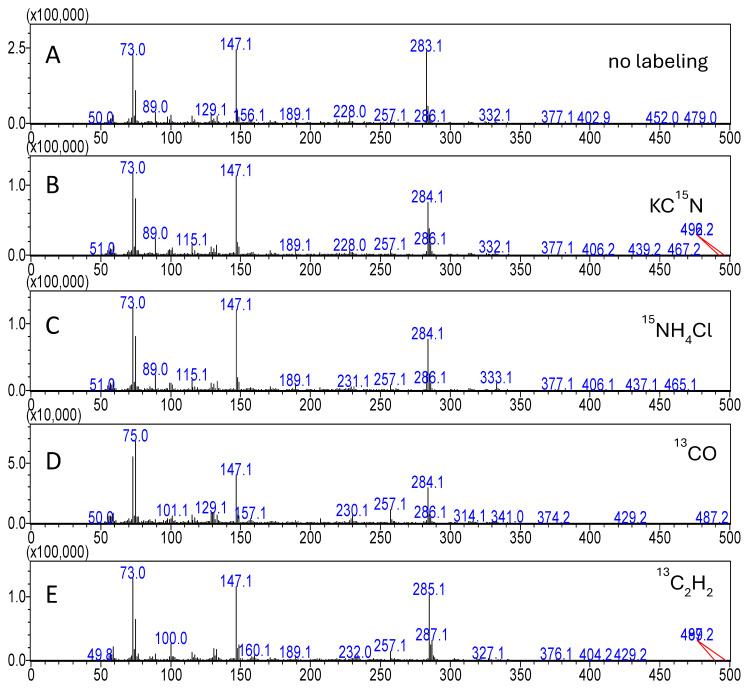
Different mass spectra of uracil. (**A**) is from run 1 (Table S1), (**B**) is from a run with KC^15^N, (**C**) is from a run with ^15^NH_4_Cl, (**D**) is from a run with ^13^CO and (**E**) is from a run with ^13^C_2_H_2_. The typical mass of *m*/*z* = 284 results from the loss of the t-Butyl group from TBDMS. The red lines only indicate an overload of the detector, but cannot be changed in the output.

**Figure 2:**
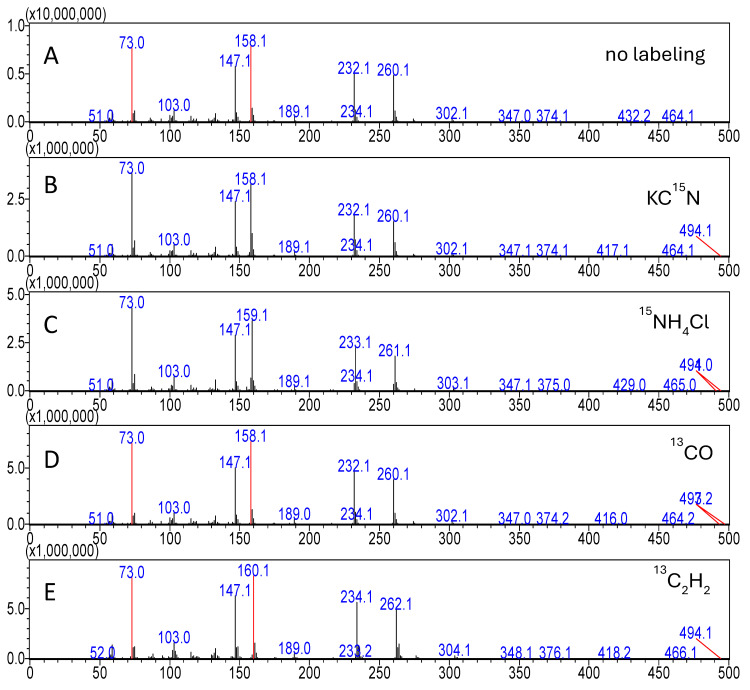
Different mass spectra of alanine. Shown are the masses of typical fragments of TBDMS-derivatized amino acids. In the case of alanine these are *m*/*z* = 260, *m*/*z* = 232 and *m*/*z* = 158. (**A**) is from run 1 (Table S1), (**B**) is from a run with KC^15^N, (**C**) is from a run with ^15^NH_4_Cl, (**D**) is from a run with ^13^CO and (**E**) is from a run with ^13^C_2_H_2_. The red lines only indicate an overload of the detector, but cannot be changed in the output.

**Figure 3:**
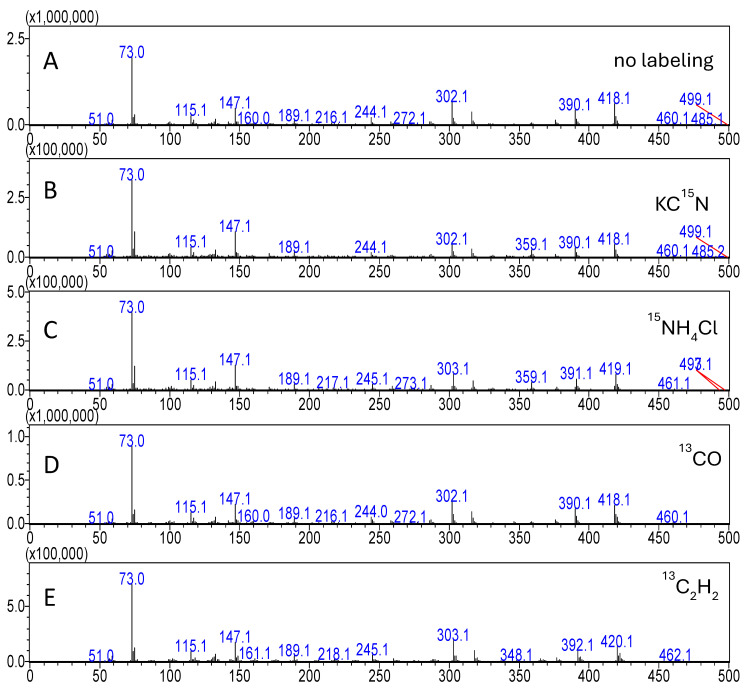
Different mass spectra of aspartic acid. (**A**) is from run 1 (Table S1), (**B**) is from a run with KC^15^N, (**C**) is from a run with ^15^NH_4_Cl, (**D**) is from a run with ^13^CO and (**E**) is from a run with ^13^C_2_H_2_. Typical masses of TBDMS-derivatized aspartic acid are *m*/*z* = 418, *m*/*z* = 390 and *m*/*z* = 302. The red lines only indicate an overload of the detector, but cannot be changed in the output.

Figure 1 shows the mass spectra of uracil, where A is an experiment without any labeled substance, B is the experiment where KC^15^N was used, C the experiments in which ^15^NH_4_Cl was added, D the experiments which were carried out with ^13^CO, and E the ones with ^13^C-acetylene. Figure 2 shows the mass spectra of alanine and Figure 3 the mass spectra of aspartic acids with the same notation as shown above.

**Scheme 1:**
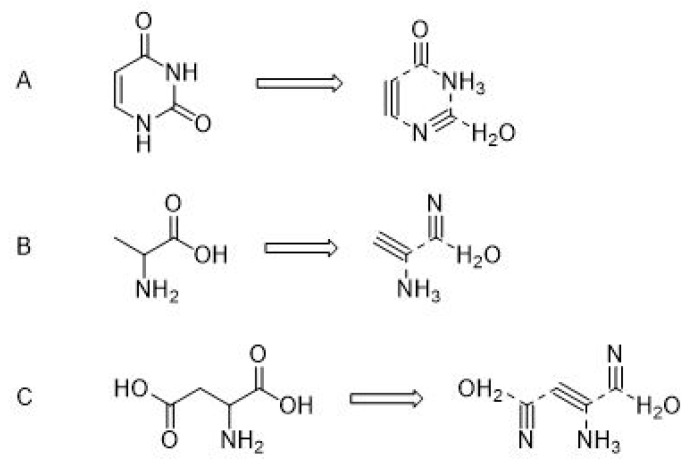
Retrosynthesis of uracil from one molecule of ammonia, acetylene, cyanide and carbon monoxide (**A**), alanine from one molecule of ammonia, one molecule of acetylene, one molecule of cyanide and one molecule of water (**B**), and aspartic acid from one molecule of ammonia and acetylene and two molecules of cyanide and water (**C**).

Mass spectra of uracil obtained from unlabeled experiments and from reactions containing labeled cyanide, ammonium, carbon monoxide, or acetylene reveal distinct incorporation patterns. These patterns demonstrate that uracil formation involves contributions from all four reactants. Comparable labeling experiments conducted for alanine and aspartic acid show that the carbon skeletons of both amino acids originate primarily from acetylene, while the amino group is derived from ammonium. Carboxyl groups are preferentially formed via cyanide hydrolysis. Based on these results, retrosynthetic pathways for all three products were reconstructed. Alanine formation requires one molecule each of acetylene, ammonia, cyanide, and water, whereas aspartic acid formation involves acetylene, ammonia, and two equivalents of cyanide and water. Uracil formation follows a distinct pathway, incorporating two OCN units derived from cyanide and from carbon monoxide and ammonia, respectively, with acetylene contributing to the remaining carbon framework (Scheme 1).

**Scheme 2:**
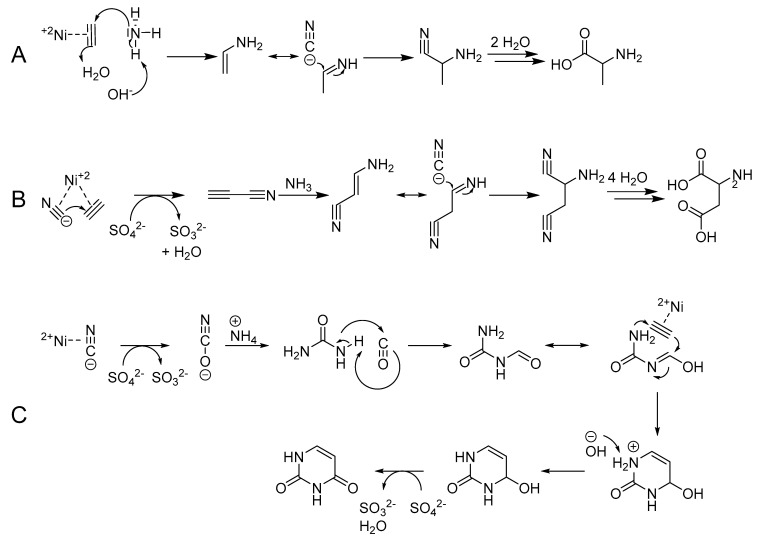
Proposed reaction mechanism of the metal catalyzed formation of alanine (**A**) aspartic acid (**B**) and uracil (**C**). Here, Ni is used as an example for the metal catalyst.

It has been demonstrated that nickel exhibits a strong affinity for acetylene [47,48]. Consequently, the prebiotic synthesis of amino acids from ammonia and cyanide is likely to proceed via a Strecker-type reaction. In the initial step, acetylene undergoes nucleophilic attack by ammonia, a process that is facilitated by coordination to a metal center. This could be followed by attack of the cyanide ion on the activated acetylene, and subsequent hydrolysis yields the corresponding carboxylic acid groups (Scheme 2A).

In the case of aspartic acid, a cyanoacetylene intermediate is presumably formed first, a transformation that is likewise promoted by nickel coordination. The remainder of the amino acid framework is then generated through the same reaction sequence described above (Scheme 2B).

The synthesis of uracil proceeds from the cyanide ion through the intermediates cyanate and urea, ultimately forming N-carbamoylformamide. This compound can subsequently react with acetylene via an oxidative-ring-closure reaction to yield uracil (Scheme 2C).

**Figure 4:**
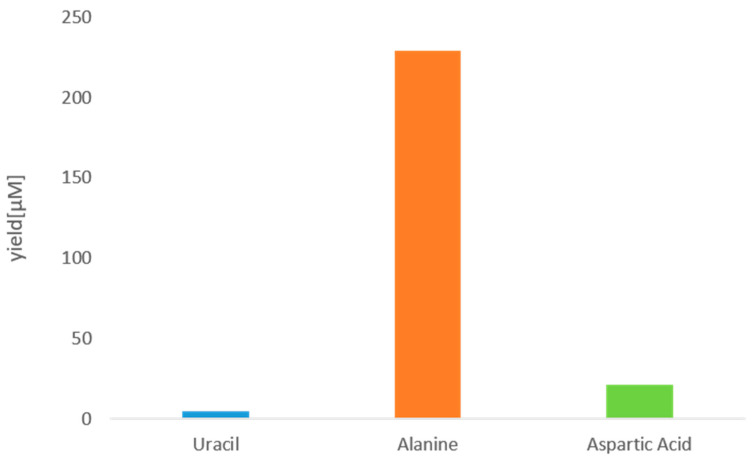
Comparison of the yield of uracil (blue), alanine (orange), and aspartic acid (green) from a typical run (Table S1) after 7 days.

Reaction parameters including catalyst identity, pH and reaction time were systematically varied. Tables S1–S3 show all reactions which were performed to see the influence of the reaction parameters. Across all conditions tested, amino acids were produced in significantly higher yields than uracil. Alanine consistently dominated the product mixture, followed by aspartic acid, whereas uracil was detected in substantially lower concentrations (Figure 4). No additional nucleobases or amino acids could be detected under the conditions employed. Under similar conditions the production of more amino acids was shown earlier [49].

As alanine dominates the product mixture, the amount of uracil was increased by a factor of 1000 and the amount of aspartic acid by a factor of 100 in the following figures for better comparability.

**Figure 5:**
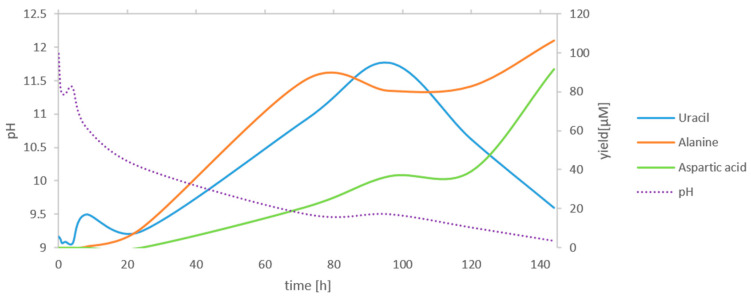
Time-dependent NiS catalyzed formation of uracil (blue), alanine (orange), and aspartic acids (green) from acetylene, carbon monoxide cyanide and ammonia.

Time-course experiments revealed distinct formation profiles for amino acids and uracil. While alanine and aspartic acid yields increased steadily over time, uracil formation exhibited a pronounced maximum after approximately 96 h, followed by a sharp decline (Figure 5). Whether this decrease results from decomposition or transformation into more complex products remains unclear.

**Figure 6:**
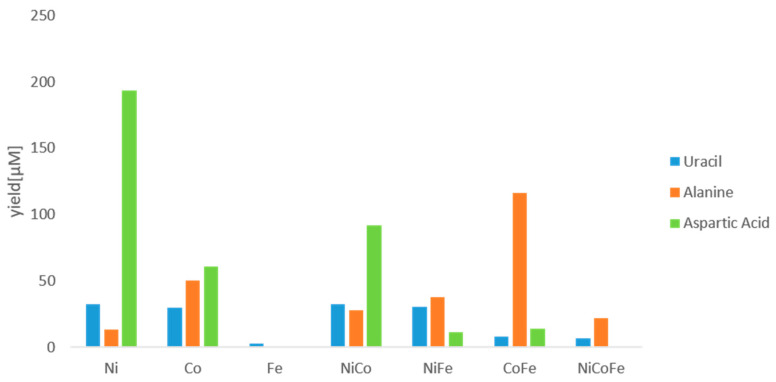
Formation of uracil (blue), alanine (orange), and aspartic acid (green) with different metal ions as metal sulfide catalysts after 7 days. Mixtures of transition metal catalysts contains 50/50 (mol%) or 33/33/33 (mol%) of the corresponding metal sulfides, respectively.

Transition metal sulfides of iron, nickel, and cobalt all catalyzed product formation. While amino acid yields were strongly dependent on the specific metal catalyst employed, uracil yields were comparatively insensitive to catalyst identity, with the exception of iron, the mixture of iron and cobalt, and the combination of all three metals, which showed reduced activity (Figure 6). No product formation was observed if the metal sulfide catalyst was omitted, showing the importance of the metal sulfide surfaces in this context.

**Figure 7:**
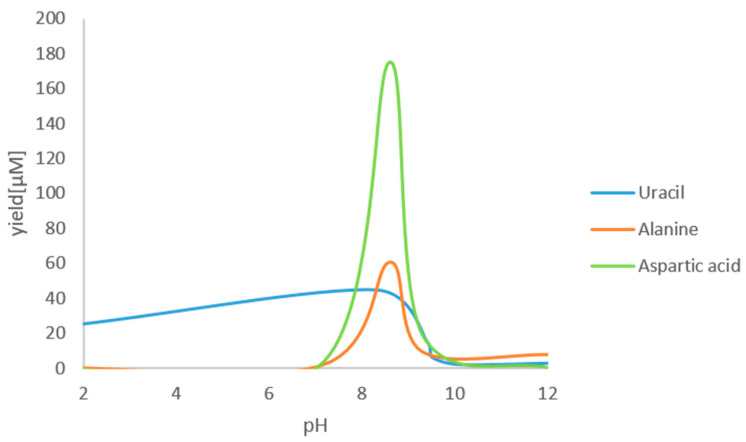
Formation of uracil (blue), alanine (orange), and aspartic acid (green) in the presence of NiS at different pH values after 7 days. pH values were measured at the end of the reaction time.

For determination of the pH dependence, aqueous NaOH, aqueous H_2_SO_4_, and solid Ca(OH)_2_ were added to the reaction settings. pH values were measured at the end of the reaction time (7 days). Optimal product formation was observed at pH values around 9, consistent with the pKa values of cyanide and ammonium and with previous studies demonstrating catalyst inhibition at higher hydroxide concentrations [43,44] (Figure 7).

## 4. Discussion

Although only the nucleobase uracil and a limited number of amino acids were formed in detectable amounts under these specific conditions, we nevertheless venture to draw conclusions from these observations. The results implicate the possibility that, under geochemically plausible iron–sulfur world conditions, molecular precursors associated with both proto-metabolic processes and early informational polymers can emerge simultaneously. Rather than resolving the dichotomy between *metabolism-first* and *information-first* frameworks in favor of one over the other, this concurrent formation supports a more integrated scenario in which both chemical systems co-evolved from the outset. Such a setting is consistent with the emergence of an already proposed RNA–peptide world [50,51,52], where rudimentary metabolic networks, short peptides, and RNA molecules interacted synergistically during the earliest stages of chemical evolution.

Although both molecular classes form under identical conditions, amino acids are produced in substantially higher yields than the nucleobase uracil. This imbalance could suggest that peptide formation may have been quantitatively favored in early environments, potentially leading to the early availability of short peptides with catalytic or stabilizing functions. Further metabolically relevant compounds were formed under related conditions, including intermediates of the citrate cycle [12], fatty acids [53], aldehydes [54], and amides [43], which can serve as precursors to longer peptide chains. Together, these observations could support a scenario in which proto-metabolic chemistry and peptide formation progressed efficiently, while nucleobases accumulated more gradually. In an RNA–peptide world context, this would imply that early peptides may have played a crucial role in facilitating or stabilizing the emergence of RNA, rather than metabolism or RNA acting independently.

Geochemically plausible environments such as hydrothermal vent systems would provide a natural setting for such co-evolution. Transition metal–sulfur minerals, as proposed in Wächtershäuser’s iron–sulfur world hypothesis, could have catalyzed key redox and carbon-fixation reactions while simultaneously supporting the formation of amino acids and nucleobases. Within these environments, spatial confinement, mineral surfaces, and energy gradients could have facilitated repeated interactions between peptides and RNA precursors.

Finally, we speculate that this framework could provide a gradual and continuous transition from geochemistry to biochemistry, consistent with non-equilibrium thermodynamics and self-organization principles. Instead of requiring the independent emergence of complex RNA or fully developed metabolic cycles, it would allow incremental increases in molecular complexity through cooperative interactions.

## Data Availability

The original contributions presented in this study are included in the article/Supplementary Material. Further inquiries can be directed to the corresponding author.

## Supplementary Materials

The following supporting information can be downloaded at: https://www.mdpi.com/article/10.3390/life16040624/s1, Table S1: Uracil, alanine and aspartic acid formation based on different metal catalysts. Table S2: Uracil, alanine and aspartic acid formation based on different reaction times. Table S3: Uracil, alanine and aspartic acid formation based on different pH values.
