# Supplementary material for: A Clue for the Hen and Egg Question: The Simultaneous Formation of Uracil and Amino Acids Under Simulated Hadean Conditions
Source: Life (Basel). 2026 Apr 8;16(4):624. doi: 10.3390/life16040624 (PMC13118113; doi:10.3390/life16040624)
Supplement: Supplementary file 1 [file life-16-00624-s001.zip › life-4210944-supplementary.pdf]

Supplement

A clue to the hen and egg question: The simultaneous formation of uracil and amino acids under simulated Hadean conditions

Christian Seitz, Denis Schuldeis, Konstantin Vogel, Wolfgang Eisenreich, Claudia Huber *

Table S 1. Uracil, alanine and aspartic acid formation based on different metal catalysts........1

Table S 2. Uracil, alanine and aspartic acid formation based on different reaction times 2

Table S 3. Uracil, alanine and aspartic acid formation based on different pH values...............2

***Table S 1**. Uracil, alanine and aspartic acid formation based on different metal catalysts. Ni, Co, Fe and mixture of them were used. Metal sulfides were formed in situ from metal sulfates and Na_2S. Other parameters as stated above. Defined standard run is marked in red (1mmol CoS catalyst).*

CoS	NiS	FeS	pH	Conc. Uracil	Conc. Alanine	Conc. Aspartic acid	Yield Uracil	Yield Alanine	Yield Aspartic acid
[mmol]	[mmol]	[mmol]		[µM]	[µM]	[µM]	[‰]	[‰]	[‰]
1			8.5	0.033	13.078	19.364	0.033	13.078	19.364
	1		8.5	0.030	50.293	6.101	0.030	50.293	6.101
		1	9.1	0.003	0.639	0.010	0.003	0.639	0.010
0.5	0.5		8.7	0.033	27.894	9.173	0.033	27.894	9.173
	0.5	0.5	8.6	0.030	37.697	1.101	0.030	37.697	1.101
0.5		0.5	8.5	0.008	116.406	1.402	0.008	116.406	1.402
0.33	0.33	0.33	8.7	0.006	21.855	0.023	0.006	21.855	0.023

Table S 2. *Uracil, alanine and aspartic acid formation based on different reaction times. Other parameters as stated above.*

time [min]	pH	Conc. Uracil [µM]	Conc. Alanine [µM]	Conc. Aspartic acid [µM]	Yield Uracil [‰]	Yield Alanine [‰]	Yield Aspartic acid [‰]
0	11.9	0.006	<0.001	<0.001	0.006	<0.001	<0.001
30	11.4	0.004	<0.001	<0.001	0.004	<0.001	<0.001
60	11.3	0.002	<0.001	<0.001	0.002	<0.001	<0.001
120	11.3	0.003	<0.001	<0.001	0.003	<0.001	<0.001
240	11.4	0.002	<0.001	<0.001	0.002	<0.001	<0.001
480	10.8	0.017	0.533	<0.001	0.017	0.533	<0.001
1440	10.2	0.008	9.865	0.002	0.008	9.865	0.002
4320	9.5	0.066	86.252	2.096	0.066	86.252	2.096
5760	9.5	0.095	80.422	3.665	0.095	80.422	3.665
7200	9.3	0.056	82.759	3.929	0.056	82.759	3.929
8640	9.1	0.020	106.168	9.162	0.020	106.168	9.162

Table S 3. *Uracil, alanine and aspartic acid formation based on different pH values , which were achieved by adding different volumes of NaOH solution, H_2SO_4 solution and solid $Ca(OH)_2$. Other parameters as stated above.*

NaOH [ml]	H2SO4 [ml]	Ca(OH)2 [g]	pH	Conc. Uracil [µM]	Conc. Alanine [µM]	Conc. Aspartic acid [µM]	Yield Uracil [‰]	Yield Alanine [‰]	Yield Aspartic acid [‰]
0			8.3	0.025			0.025		
1			9.4	0.045			0.045		
1.5			9.5	0.015			0.015		
2.5			10.1	0.006			0.006		
	1		1.9	0.002			0.002		
		0.5	12.7	0.003			0.003		
0			7.1		0.915	0.162		0.915	0.162
1			8.9		1.524	0.201		1.524	0.201
1.5			9.3		60.718	17.487		60.718	17.487
2.5			10.1		9.586	1.963		9.586	1.963
	2		0.9		8.006	0.009		8.006	0.009
